# Public assistance and survival equality in patients with *EGFR* mutation-positive lung cancer

**DOI:** 10.1093/jjco/hyae167

**Published:** 2024-12-01

**Authors:** Kiyoaki Uryu, Yoshinori Imamura, Rai Shimoyama, Takahiro Mase, Yoshiaki Fujimura, Maki Hayashi, Megu Ohtaki, Keiko Otani, Makoto Hibino, Shigeto Horiuchi, Tomoya Fukui, Ryuta Fukai, Yusuke Chihara, Akihiko Iwase, Noriko Yamada, Yukihiro Tamura, Hiromasa Harada, Asuka Tsuya, Takafumi Okabe, Masahiro Fukuoka, Hironobu Minami

**Affiliations:** Department of Medical Oncology, Yao Tokushukai General Hospital, Yao-shi, Osaka 581-0011, Japan; Cancer Care Promotion Center, University of Fukui Hospital, Yoshida-gun, Fukui 910-1193, Japan; Department of General Surgery, Shonan Kamakura General Hospital, Kamakura-shi, Kanagawa 247-8533, Japan; Department of Breast and Endocrine Surgery, Ogaki Tokushukai Hospital, Ogaki-shi, Gifu 503-0015, Japan; Tokushukai Information System Inc., Kita-ku, Osaka 530-0001, Japan; Mirai Iryo Research Center Inc., Chiyoda-ku, Tokyo 102-0083, Japan; deCult Co., Ltd., Hatsukaichi-shi, Hiroshima 739-0413, Japan; deCult Co., Ltd., Hatsukaichi-shi, Hiroshima 739-0413, Japan; Department of Respiratory Medicine, Shonan Fujisawa Tokushukai Hospital, Fujisawa-shi, Kanagawa 251-0041, Japan; Department of Respiratory Medicine, Shonan Fujisawa Tokushukai Hospital, Fujisawa-shi, Kanagawa 251-0041, Japan; Department of Respiratory Medicine, Shonan Kamakura General Hospital, Kamakura-shi, Kanagawa 247-8533, Japan; Department of General Thoracic Surgery, Shonan Kamakura General Hospital, Kamakura-shi, Kanagawa 247-8533, Japan; Department of Respiratory Medicine, Uji Tokushukai Medical Center, Uji-shi, Kyoto 611-0041 Japan; Department of Respiratory Medicine, Chibanishi General Hospital, Matsudo-shi, Chiba 270-2251, Japan; Department of General Thoracic Surgery, Chibanishi General Hospital, Matsudo-shi, Chiba 270-2251, Japan; Department of General Internal Medicine, Oosumi Kanoya Hospital, Kanoya-shi, Kagoshima 893-0015, Japan; Department of Respiratory Medicine, Yao Tokushukai General Hospital, Yao-shi, Osaka 581-0011, Japan; Department of Medical Oncology, Izumi City General Hospital, Izumi-shi, Osaka 594-0073, Japan; Department of Medical Oncology, Izumi City General Hospital, Izumi-shi, Osaka 594-0073, Japan; Department of Medical Oncology, Izumi City General Hospital, Izumi-shi, Osaka 594-0073, Japan; Department of Medical Oncology and Hematology, Kobe University Graduate School of Medicine, Chuo-ku, Kobe-shi 650-0017, Japan; Cancer Center, Kobe University Hospital, Chuo-ku, Kobe-shi 650-0017, Japan

**Keywords:** non-small cell lung cancer, epidermal growth factor receptor tyrosine kinase inhibitor, disparity, public assistance, urbanization level

## Abstract

**Background:**

Disparities in public assistance or the urbanization level of a residential region can affect cancer treatment outcomes. This study aimed to investigate whether these factors affect the overall survival (OS) of patients with epidermal growth factor receptor (*EGFR*) mutation-positive non-small cell lung cancer (NSCLC) using Tokushukai REAL World Data.

**Methods:**

We analyzed the clinical data of consecutive patients with NSCLC receiving EGFR-tyrosine kinase inhibitors between April 2010 and March 2020 at 46 Tokushukai Medical Group hospitals in Japan. The patient’s insurance coverage status was extracted from electronic medical records, and the urbanization level of residential regions was classified as megalopolis or other according to the secondary medical region. Univariate and multivariate Cox regression analyses were performed to examine the associations between OS and patient/tumor/treatment/socioeconomic-related factors.

**Results:**

In total, 758 patients (58.5% females) were included in the study; 41 patients (5.4%) received public assistance, and 442 patients (58.3%) were categorized under megalopolis in the secondary medical regions. In multivariate Cox regression analyses, there was no significant difference in the OS between non-recipients of public assistance and recipients [hazard ratio (HR) 1.084; 95% confidence intervals (CIs), 0.674–1.744]. There was also no significant difference in the OS between megalopolis and other regions in the secondary medical regions (HR 1.143; 95% CIs, 0.914–1.428).

**Conclusions:**

Our findings suggest that neither the use of public assistance nor the urbanization level in the residential region significantly impacts the prognosis of Japanese patients with *EGFR* mutation-positive NSCLC.

## Teaser

Considering the impact of disparity on the OS in patients with *EGFR* mutation-positive NSCLC, recipients of public assistance and residents of non-megalopolis regions also received secured treatment in Japan.

## Introduction

Recent studies indicate that disparities related to gender, race, or socioeconomic status affect cancer treatment outcomes [1–3]. To address these disparities, medical policies are developed, acknowledging that healthcare systems vary greatly by country and region. The public assistance system is designed to support individuals in leading a healthy lifestyle and has been implemented in several countries, each with unique characteristics.

In Japan, ‘public assistance’ refers to the Livelihood Protection System, which ensures access to medical care for individuals with low income—funded by the Ministry of Health, Labour and Welfare. The most notable feature is its universal healthcare system, which has been in place since 1950 [4]. Residents can receive most of the standard therapies, with out-of-pocket expenses varying between 10% and 30% depending on age and income. On the other hand, individuals receiving public assistance are excluded from national health insurance, with the majority of their medical expenses being fully covered by the local government. This is because Japan’s public assistance system is based on the principle of providing necessary support according to the degree of need, ensuring a minimum standard of health and cultural living, while also promoting self-reliance [5]. As of 2022, there were ~2.04 million recipients of public assistance in Japan, accounting for 1.63% of the population [5]. An increase in the number of public assistance recipients places a significant strain on Japan’s healthcare economy. Economic downturns, such as those experienced during the COVID-19 pandemic, increase enrollment in public assistance programs, which, in turn, increases healthcare demand. This places pressure on local government budgets, potentially resulting in cuts in medical services and a decline in the quality of care [6,7].

Even under a universal healthcare system, public assistance has been identified as an additional risk factor for the development of congestive heart failure, chronic obstructive pulmonary disease, chronic renal failure, and diabetes [8,9]. However, its impact on cancer care has not been fully elucidated [10].

In Japan, prefectures have established primary, secondary, and tertiary medical regions to formulate medical policies [11]. Primary regions focus on routine care through clinics and are usually centered around municipalities. Secondary regions offer general inpatient care, including emergency and cancer treatment. They are typically comprised of multiple municipalities, taking into account factors such as population size and fluctuations in patient numbers. There are 344 of these regions, and they are fundamental for planning the number of doctors and hospital beds. Tertiary regions provide specialized care, such as severe burns and organ transplants, and generally consist of entire prefectures. As the government transitions from a ‘hospital-centered’ to a ‘community-centered’ healthcare system, it is crucial to examine whether this shift has equalized cancer care. Although several studies have reported that urbanization affects the quality of cancer treatment [11], Japanese data on whether this transition has balanced care across different regions are limited [12].

In this study, a supplementary analysis was performed using data from a Japanese real-world study [13] to compare overall survival (OS) in lung cancer treatment between recipients and non-recipients of public assistance and evaluate the impact of the urbanization level of the residential area.

## Materials and methods

### Study populations

This nationwide retrospective cohort study was conducted in compliance with ethical guidelines and as part of the Tokushukai REAl-World Data (TREAD) project. This study was approved by the Institutional Review Board of the Tokushukai Medical Group (TGE01427–024), registered with the UMIN Clinical Trial Registry (UMIN000050590). Due to the retrospective nature of the study, the requirement for written informed consent was waived [14].

We evaluated patients with advanced or recurrent non-small cell lung cancer (NSCLC) who were treated with epidermal growth factor receptor (EGFR)-tyrosine kinase inhibitors (TKIs) as first-line palliative therapy at the Tokushukai Medical Group hospitals. This network comprises 46 hospitals across Japan with a total of 14 829 beds. These hospitals used a shared medical record system (e-Karte and Newtons2; Software Service Inc., Osaka, Japan) and a chemotherapy protocol system (srvApmDrop; Software Service Inc., Osaka, Japan) between 1 April 2010 and 31 March 2020. Patients with active double cancer, without a complete treatment history, or with unknown or wild-type *EGFR* mutations were excluded from this study [13].

### Data collection

Data for patients fulfilling the inclusion criteria were extracted by Tokushukai Information System Inc., with researchers blinded to the statistical analyses. Demographic information—including age, birth year and month, sex, postal code, and insurance type—was recorded at the time of medical record identification. Additionally, body height, weight, body mass index, body surface area, and corresponding dates of measurement were documented as required for clinical purposes. Furthermore, dates of the last visit, last survival confirmation, death, and diagnoses on medical receipts were automatically recorded as part of routine clinical practice. This information is retrievable from a unified medical record system (e-Karte and Newtons2, Software Service, Inc., Osaka, Japan) [14].

The explanatory variables at the time of first-line EGFR-TKI initiation [including sex, age, body mass index (BMI), smoking status, disease status, *EGFR* mutation status, first-line EGFR-TKI, insurance coverage status, and urbanization level of residential region], details of NSCLC treatments (including EGFR-TKIs, other systemic agents, surgery, and radiotherapy), and prognosis (final date of survival confirmation, date of death, cause of death) were extracted from the medical record system, chemotherapy protocol system, and National Cancer Registry Data in Japan [15].

Recipients of public assistance were extracted from the insurance coverage of receipts during the treatment period. The urbanization level of the residential region was classified by population and population density (megalopolis, population ≥ 1 million people or population density ≥ 2000 persons/km^2^; urban, population ≥ 200 000 and < 1 million and population density < 2000 persons/km^2^, or population ≥ 100 000 with a population density ≥ 200 persons/km^2^ but <2000 persons/km^2^; rural, regions that were neither megalopolis nor urban), according to the secondary medical regions. With this classification, the 344 secondary medical regions in Japan could be categorized into 52 metropolitan, 172 urban, and 120 rural [11].

Identifiable information such as names, addresses, and contact details was removed from the dataset. Additionally, data handling and storage complied with the local regulations on patient confidentiality and the Data Protection Act.

### Statistical analysis

Survival analysis of OS was performed using Cox regression methods. The explanatory variables included in the model were selected based on their clinical relevance and established significance in the literature concerning OS in *EGFR* mutation-positive NSCLC. These variables included age, sex, performance status, smoking history, treatment regimen (EGFR-TKIs), and socioeconomic factors, such as insurance type and urbanization level. OS represented the duration from the initiation of EGFR-TKI therapy to death due to any cause. The censored cases included patients who were alive at the end date of the study or who dropped out of the study for any reason. Cox regression analysis was performed using all the prognostic factors, referred to as the full model. Model selection was performed using the Akaike information criterion (AIC) [16], which involves determining the combination of variables that yields the smallest AIC while considering the significance of the explanatory variables and the AIC value. The model consisting of these prognostic factors is referred to as the optimal model.

A stratified Cox proportional model was used to obtain adjusted Kaplan–Meier survival curves of a prognostic factor of interest, and its significance (null hypothesis: the item was not involved in the goodness of fit of the model) was obtained using a likelihood ratio test. The analyses were conducted using R, version 4.2.2, from the R Foundation for Statistical Computing, Vienna, Austria. All statistical evaluations were two-sided, and significance was set at a threshold of <0.05.

## Results

### Patient characteristics

In total, 758 patients with *EGFR* mutation-positive advanced NSCLC were included in this study [13]. Of those, 41 (5.4%) were recipients of public assistance. Table 1a summarizes the baseline demographic and clinical characteristics of first-line EGFR-TKIs. Smoking status showed a trend toward more smoking history among recipients of public assistance (Table 1a). The urbanization level of the residential regions according to the secondary medical regions consisted of a megalopolis, accounting for 442 (58.3%), and the remaining 316 (41.7%), of which 274 (36.2%) were urban and 42 (5.5%) were rural (Table 1b).

**Table 1a:** Patients’ characteristics by insurance

Characteristics	Category	Non-public assistance recipients *N* = 717 (94.6%)	Public assistance recipients *N* = 41 (5.4%)	*P*-value (χ2-test/Fisher’s exact test)
Sex	Females	423 (59.0)	21 (51.2)	0.412
	Males	294 (41.0)	20 (48.8)	
Age (years)	<80	530 (73.9)	32 (78.0)	0.686
	≥80	187 (26.1)	9 (22.0)	
ECOG-PS	0	71 (9.9)	4 (9.7)	0.583^a^
	1	199 (27.7)	12 (29.3)	
	≥2	50 (7.0)	5 (12.2)	
	NA	397 (55.4)	20 (48.8)	
Body mass index	< 25	628 (87.6)	31 (75.6)	0.252^a^
	≥ 25	67 (9.3)	6 (14.6)	
	NA	22 (3.1)	4 (9.8)	
Smoking status	Current or former	143 (19.9)	18 (43.9)	0.000^a^
	Never	529 (73.8)	21 (51.2)	
	NA	45 (6.3)	2 (4.9)	
Histology	Adenocarcinoma	665 (92.7)	36 (87.8)	0.225
	Others	52 (7.3)	5 (12.2)	
Disease status	Advanced/inoperable	77 (10.7)	6 (14.6)	0.609
	Metastatic	494 (68.9)	28 (68.3)	
	Recurrent	146 (20.4)	7 (17.1)	
EGFR mutational status	Del 19	302 (42.1)	12 (29.3)	0.283
	L858R	291 (40.6)	24 (58.5)	
	T790M	41 (5.7)	1 (2.4)	
	Others	56 (7.8)	3 (7.3)	
	Positive (details unknown)	27 (3.8)	1 (2.4)	
Secondary medical region	Megalopolis	423 (59.0)	19 (46.3)	0.151
	Others	294 (41.0)	22 (53.7)	

^a^The test does not include ‘NA’.

**Table 1b:** Patients’ characteristics by medical regions

Characteristics	Category	Megalopolis *N* = 442 (58.3%)	Others *N* = 316 (41.7%)	*P*-value (χ2-test/Fisher’s exact test)
Sex	Females	258 (58.4)	186 (58.9)	0.952
	Males	184 (41.6)	130 (41.1)	
Age (years)	<80	326 (73.8)	236 (74.7)	0.839
	≥80	116 (26.2)	80 (25.3)	
ECOG-PS	0	47 (10.6)	28 (8.9)	0.559^a^
	1	145 (32.8)	66 (20.9)	
	≥2	35 (7.9)	20 (6.3)	
	NA	215 (48.6)	202 (64.0)	
Body mass index	< 25	384 (86.9)	275 (87.0)	0.439^a^
	≥ 25	40 (9.0)	33 (10.4)	
	NA	18 (4.1)	8 (2.5)	
Smoking status	Current or former	101 (22.9)	60 (19.0)	0.425^a^
	Never	315 (71.3)	235 (74.4)	
	NA	26 (5.9)	21 (6.6)	
Histology	Adenocarcinoma	356 (80.5)	258 (81.6)	0.774
	Others	86 (19.5)	58 (18.5)	
Disease status	Advanced/inoperable	54 (12.3)	29 (9.2)	0.225
	Metastatic	306 (69.2)	216 (68.4)	
	Recurrent	82 (18.5)	71 (22.5)	
EGFR mutational status	Del 19	183 (41.4)	131 (41.5)	0.401
	L858R	183 (41.4)	132 (41.8)	
	T790M	23 (5.2)	19 (6.0)	
	Others	40 (9.0)	19 (6.0)	
	Positive (details unknown)	13 (2.9)	15 (4.7)	
Insurance	Public assistance recipients	19 (4.3)	22 (7.0)	0.151
	Non-public assistance recipients	423 (95.7)	294 (93.0)	

^a^The test does not include ‘NA’.

### Cancer treatments

Cancer treatments for the 758 patients are summarized in Table 2a by public assistance, and in Table 2b by urbanization level. In both system groups, gefitinib was the most frequently used EGFR-TKI. The proportion of patients who received afatinib tended to be higher among non-recipients of public assistance, whereas the proportion of patients who received erlotinib tended to be higher among recipients of public assistance. No patients in our cohort received dacomitinib at any time. The median number of EGFR-TKIs administered was 1 (range, 1–4) in both system groups. During treatment, 6.0% of the non-recipients of public assistance and 2.4% of the recipients of public assistance received systemic chemotherapy with immune checkpoint inhibitors other than EGFR-TKIs. None of the differences were significant according to public assistance (Table 2a). Erlotinib was used more frequently in megalopolis and osimertinib less frequently. During treatment, 3.8% of the megalopolis and 8.5% of the other regions received systemic chemotherapy with immune checkpoint inhibitors (Table 2b).

**Table 2a:** Summary of cancer treatment by insurance

Characteristics	Category	Non-public assistance recipients *N* = 717 (94.6%)	Public assistance recipients *N* = 41 (5.4%)	*P*-value (χ2-test/Fisher’s exact test)
Prior surgery	Curative	90 (12.6)	4 (9.8)	0.233^a^
	Palliative	21 (2.9)	3 (7.3)	
	None	499 (69.6)	28 (68.3)	
	NA	107 (14.9)	6 (14.6)	
Prior radiotherapy	Curative	26 (3.6)	0	0.729^a^
	Palliative	57 (7.9)	2 (4.9)	
	None	526 (73.4)	33 (80.5)	
	NA	108 (15.1)	6 (14.6)	
Prior systemic therapy, other than EGFR-TKI	Platinum agents	76 (10.6)	8 (19.5)	0.502
	Taxanes	28 (3.9)	3 (7.3)	
	Angiogenesis inhibitors	20 (2.8)	4 (9.8)	
	Other cytotoxic agents	4 (0.6)	1 (2.4)	
First-line EGFR-TKI	Gefitinib	388 (54.1)	23 (56.1)	0.325
	Erlotinib	98 (13.7)	9 (22.0)	
	Afatinib	101 (14.1)	3 (7.3)	
	Osimertinib	130 (18.1)	6 (14.6)	
Sequential systemic therapy, other than EGFR-TKI	Platinum agents	134 (18.7)	6 (14.6)	0.179
	Taxanes	84 (11.7)	3 (7.3)	
	Angiogenesis inhibitors	95 (13.2)	3 (7.3)	
	Immune checkpoint inhibitors	43 (6.0)	1 (2.4)	
	Other cytotoxic agents	15 (2.1)	3 (7.3)	

^a^The test does not include ‘NA’.

**Table 2b:** Summary of cancer treatment by medical regions

Characteristics	Category	Megalopolis *N* = 442 (58.3%)	Others *N* = 316 (41.7%)	*P*-value (χ2-test/Fisher’s exact test)
Prior surgery	Curative	52 (11.8)	42 (13.3)	0.541^a^
	Palliative	14 (3.2)	10 (3.2)	
	None	323 (73.1)	204 (64.6)	
	NA	53 (12.0)	60 (19.0)	
Prior radiotherapy	Curative	12 (2.7)	14 (4.4)	0.116
	Palliative	31 (7.0)	28 (8.9)	
	None	346 (78.3)	213 (67.4)	
	NA	53 (12.0)	61 (19.3)	
Prior systemic therapy, other than EGFR-TKI	Platinum agents	46 (10.4)	38 (12.0)	0.11
	Taxanes	24 (5.3)	7 (2.2)	
	Angiogenesis inhibitors	14 (3.2)	10 (3.2)	
	Other cytotoxic agents	2 (0.4)	3 (0.9)	
First-line EGFR-TKI	Gefitinib	241 (54.5)	170 (53.8)	0.03
	Erlotinib	73 (16.5)	34 (10.8)	
	Afatinib	61 (13.8)	43 (13.6)	
	Osimertinib	67 (15.2)	69 (21.8)	
Sequential systemic therapy, other than EGFR-TKI	Platinum agents	95 (21.5)	45 (14.2)	0.014
	Taxanes	53 (12.0)	34 (10.8)	
	Angiogenesis inhibitors	63 (14.3)	35 (11.1)	
	Immune checkpoint inhibitors	17 (3.8)	27 (8.5)	
	Other cytotoxic agents	11 (2.5)	7 (2.2)	

^a^The test does not include ‘NA’.

### Cox regression and Kaplan–Meier analyses

#### Comparison by insurance system

**Figure 1:**
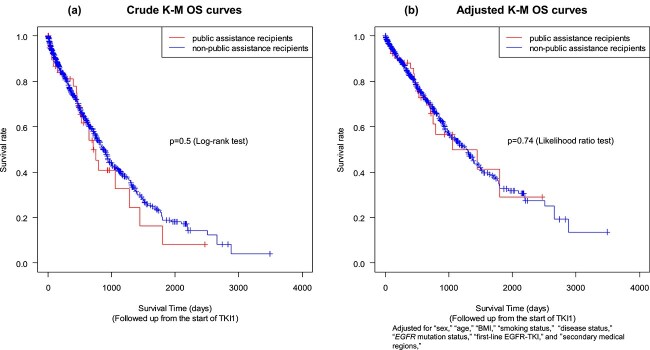
Kaplan–Meier survival curves. Crude Kaplan–Meier survival curves for recipients and non-recipients of public assistance are shown in (a), and adjusted Kaplan–Meier survival curves for sex, age, body mass index, smoking status, disease status, *EGFR* mutation status, first-line EGFR-tyrosine kinase inhibitors, and secondary medical region are shown in (b). No significant differences are observed between recipients and non-recipients of public assistance in either the crude or adjusted Kaplan–Meier curves.

With a median follow-up period for all patients of 15.8 months, the median crude Kaplan–Meier survival for non-recipients and recipients of public assistance was 29.1 months [95% confidence interval (CI), 25.9–31.5] and 23.7 months (95% CI, 17.8–59.9), respectively (Fig. 1a). Although the intention was to incorporate all initially relevant factors into the Cox regression model, ECOG-PS was subsequently excluded from both the univariate and multivariate analyses because of a significant amount of missing data (55.0%, as indicated in Tables 1a and 1b). Therefore, the final set of factors included in the analysis consisted of ‘sex,’ ‘age,’ ‘BMI,’ ‘smoking status,’ ‘disease status,’ ‘*EGFR* mutation status,’ ‘first-line EGFR-TKI,’ and ‘urbanization level.’ Adjusted Kaplan–Meier survival curves for non-recipients and recipients of public assistance are shown in Fig. 1b. The median adjusted Kaplan–Meier survival for non-recipients and recipients of public assistance was 43.2 months (95% CI, 30.0–83.7) and 35.2 months (95% CI, 23.7–inf.), respectively. There was no significant difference in OS between non-recipients and recipients of public assistance. Crude Kaplan–Meier survival curves by EGFR-TKI for non-recipients and recipients of public assistance are shown in Fig. S1. Osimertinib predominantly contributed to better OS regardless of public assistance being received.

Estimated hazard ratio (HR) with 95% CI and their p-values for each category of OS-related prognostic factors in the univariate and multivariate analysis are shown in Table 3. Multivariate analysis showed no difference in survival between non-recipients and recipients of public assistance (HR, 1.084; 95% CI, 0.674–1.744).

**Table 3:** Estimated HRs due to univariate and multivariate Cox regression analysis of prognostic factors for OS

		Univariate analysis				Multivariate analysis			
Item	Category	HR	L95%	U95%	*P*-value	HR	L95%	U95%	*P*-value
Sex	Females (ref.)	1.000			[0.21]	1.000			[0.208]
	Males	1.142	0.928	1.404	0.210	1.169	0.918	1.489	0.206
Age (years)	< 80 (ref.)	1.000			[0.005]	1.000			[0.011]
	≥ 80	1.143	1.119	1.783	0.004	1.399	1.086	1.803	0.009
BMI	< 25 (ref.)	1.000			[0.001]	1.000			[0.125]
	≧ 25	0.521	0.344	0.790	0.002	0.580	0.374	0.899	0.015
Smoking status	Never (ref.)	1.000			[0.184]	1.000			[0.999]
	Current or former	1.185	0.926	1.515	0.177	1.145	0.863	1.518	0.347
Disease status	Advantage/inoperable (ref.)	1.000			[0.000]	1.000			[0.000]
	Metastatic	1.576	1.081	2.300	0.018	1.514	1.011	2.266	0.044
	Recurrent	0.816	0.528	1.261	0.360	0.782	0.491	1.244	0.299
EGFR mutational status	Del 19 (ref.)	1.000			[0.008]	1.000			[0.012]
	L858R	1.221	0.968	1.539	0.091	1.039	0.808	1.336	0.765
	T790M	1.077	0.727	1.597	0.711	0.913	0.612	1.361	0.654
	Others	2.038	1.414	2.935	0.000	1.897	1.286	2.798	0.001
	Positive (details unknown)	1.389	0.785	2.456	0.259	1.701	0.897	3.227	0.104
EGFR-TKI	Gefitinib (ref.)	1.000			[0.000]	1.000			[0.000]
	Erlotinib	1.341	1.032	1.743	0.028	1.253	0.949	1.656	0.112
	Afatinib	0.642	0.454	0.908	0.012	0.622	0.428	0.904	0.013
	Osimertinib	0.455	0.258	0.803	0.007	0.484	0.273	0.859	0.013
Insurance	Non-public assistance recipients (ref.)	1.000			[0.484]	1.000			[0.741]
	Public assistance recipients	1.717	0.761	1.802	0.474	1.084	0.674	1.744	0.738
Secondary medical region	Megalopolis (ref.)	1.000			[0.636]	1.000			[0.243]
	Others	0.951	0.772	1.171	0.636	1.143	0.914	1.428	0.241

#### Comparison by secondary medical regions

**Figure 2:**
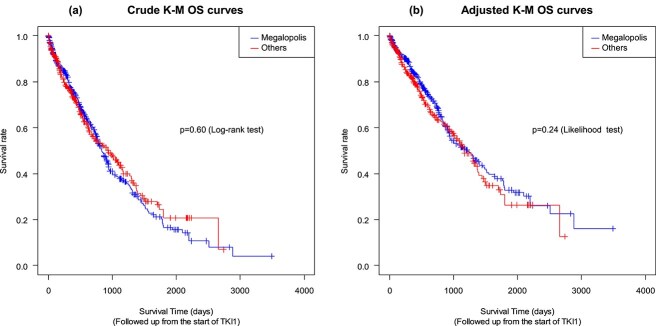
Kaplan–Meier survival curves. Crude Kaplan–Meier survival curves for secondary medical region are shown in (a), and adjusted Kaplan–Meier survival curves for sex, age, body mass index, smoking status, disease status, *EGFR* mutation status, first-line EGFR-tyrosine kinase inhibitors, and public assistance are shown in (b). Both crude and adjusted Kaplan–Meier curves are not significantly different between the secondary medical regions of megalopolis and others.

The median crude OS for the megalopolis group was 28.0 months (95% CI, 25.5–31.3), and that for other regions was 31.3 months (95% CI, 23.5–38.7; Fig. 2a). Adjusted Kaplan–Meier survival curves for sex, age, BMI, smoking status, disease status, *EGFR* mutation status, first-line EGFR-TKI, and public assistance are shown in Fig. 2b. The median adjusted OS was 40.8 months (95% CI, 28.2–83.7) for the megalopolis group, and 38.9 months (95% CI, 26.8-inf.) for other regions. There were no apparent differences in OS between megalopolis and other regions. The crude Kaplan–Meier survival curves by EGFR-TKI for megalopolis and other regions are shown in Fig. S2. Osimertinib was a major contributor to better OS regardless of the urbanization level.

Multivariate analysis showed no differences in survival between patients in megalopolis and other regions (HR, 1.143; 95% CI, 0.914–1.418; Table 3).

## Discussion

In this large retrospective cohort study, data from >750 patients with *EGFR* mutation-positive NSCLC, obtained from a shared hospital electronic medical record system covering the period from 2010 to 2020, showed no significant difference in OS between non-recipients and recipients of public assistance or residents of megalopolis and other regions. These findings suggest that recipients of public assistance and residents of non-megalopolis regions had access to secure treatment for patients with *EGFR* mutation-positive NSCLC in Japan. Regarding OS with first-line EGFR-TKIs, both the non-recipient and recipient groups experienced better OS with newer generations of EGFR-TKIs, with patients receiving osimertinib at a comparable rate for the most favorable trend, consistent with previous studies [13,17–21]. Moreover, the results suggest that Japan’s cancer care policy, based on secondary medical regions, has demonstrated some effectiveness and that significant disparities were not observed among residents of non-megalopolis regions. Neither hospital volume (defined by registered cases) nor hospital type (government-designated cancer hospital, prefectural-designated cancer hospital, or other general hospitals) were significant factors in this study as previously reported [13]. Although the lack of a significant difference in OS based on these social factors did not necessarily indicate uniformity in medical care, a minimal equity level has been achieved from a public health perspective.

Studies comparing non-recipients and recipients of public assistance with myocardial infarction have reported decreased OS in recipients of public assistance. Factors related to post-treatment lifestyle habits are considered the causes of decreased OS [22,23]. Additionally, a lower socioeconomic status has been associated with a higher short-term mortality risk, even under a universal healthcare system, attributed to lower rates of percutaneous coronary intervention after diagnosis and higher incidences of comorbidities, such as congestive heart failure, chronic obstructive pulmonary disease, and chronic renal failure [8]. Public assistance has also been identified as an additional risk factor for the development of diabetes, a lifestyle disease [9]. In this study, the reason that lung cancer treatment did not affect OS could be that, unlike lifestyle-related diseases, the impact of cancer treatment on the disease is greater than that of lifestyle factors in the case of cancer. This is supported by the similar OS despite the significantly higher smoking rates among recipients of public assistance in this study. Although *EGFR* mutation-positive NSCLC was more common among non-smokers, 49.3% of recipients of public assistance in this study were current or former smokers (Table 1a). The high smoking rate among recipients of public assistance might be attributed to differences in living environments compared with non-recipients. This study demonstrated that, in the treatment of patients with advanced/recurrent *EGFR* mutation-positive NSCLC, treatment interventions were not biased against recipients of public assistance. This indicates that the Japanese public assistance system was effective and that there was no resistance or prejudice against the treatment interventions provided for patients receiving public assistance.

The classification of secondary medical regions used in Japan divided three regions into megalopolis, urban, and rural categories based on population size and density [11]. To our knowledge, this is the first study examining how differences in urbanization impact cancer care in Japan, while only one study has investigated the influence of regional differences on the initiation of cancer treatment [24]. The Tokushukai group comprises 75 hospitals nationwide all over Japan. However, only 11 rural hospitals (26.1%) participated in this study and registered 42 patients (5.5%), which reflects a relatively low number of patients. This is likely due to the requirement that participating hospitals simultaneously meet the criteria of being part of the National Cancer Registry and designated Diagnosis Procedure Combination hospitals [5]. Therefore, in our study, urban and rural regions were combined and compared to megalopolis (Table S1). No differences in OS were observed between the two groups of megalopolis and other regions. At similar proportions, OS for patients receiving osimertinib demonstrated the most favorable trend once again (Fig. S2), in line with previous studies [13,17–21]. These findings indicate that Japan’s cancer care policy based on secondary medical regions was effective and that there were no disparities among residents of non-megalopolis regions.

This study has some limitations. First, this study is retrospective, and relevant data on recipients of public assistance and urbanization levels may not be fully available. There were relatively few recipients of public assistance and rural residents. The fact that a significant number of patients receiving public assistance were treated by the Tokushukai Group—5.4%, which is three times the national average—was not a coincidence, but rather aligned with the organization’s founding goal of achieving social equity. In this context, we believe that we secured a sufficient number of individuals to conduct a minimal yet meaningful analysis (Fig. 1, Table ,1a, and Table 2a). Second, in this study, hospitals were selected based on the Diagnosis Procedure Combination system, which may introduce selection bias. While treatment protocols are standardized within a single medical corporation group, individual differences, such as patients’ comorbidities, may still influence the outcomes. Additionally, because this study focused solely on patients who received EGFR-TKIs, whether all individuals were equitably tested for *EGFR* mutations or appropriately administered EGFR-TKIs remains unclear. Third, we did not examine racial or ethnic disparities. Historically, the Japanese population is relatively homogeneous in terms of racial and ethnic characteristics, which is why our data sources do not include identifiers for these categories. Although diversity has been increasing in recent years, under Japan’s universal healthcare system, disparities in health insurance coverage based on racial or ethnic differences are likely minimized [25]. Despite these limitations, one strength is that the national system utilizes unified electronic medical records across hospitals nationwide, enabling more accurate predictions of healthcare, welfare, and caregiving equities. Last, no economic evaluation was conducted. A recent study estimated the cost of osimertinib per month was 1.8–6.8 times higher than other TKIs [26]. It may be necessary to evaluate the economic impact of osimertinib on health care.

In conclusion, our real-world study provides valuable data on the impact of public assistance and urbanization level in the residential region on the survival of patients with *EGFR* mutation-positive NSCLC, suggesting that neither factor adversely affects patient survival. Future studies should investigate whether socioeconomic disparities exist in other types of cancer or cancer treatments as well.

## Supplementary Material

Figure_S1_2024_11_26_hyae167

Figure_S2_2024_11_26_hyae167

Table_S1_2024_11_26_hyae167

## References

[1] Aldrighetti C, Niemierko A, Van Allen E, et al. Racial and ethnic disparities among participants in precision oncology clinical studies. JAMA Netw Open 2021;4:e2133205. 10.1001/jamanetworkopen.2021.33205. 34748007 PMC8576580

[2] Duma N, Vera Aguilera J, Paludo J, et al. Representation of minorities and women in oncology clinical trials: Review of the past 14 years. J Oncol Pract 2018;14:e1–e10. 10.1200/JOP.2017.025288. 29099678

[3] Loree J, Anand S, Dasari A, et al. Disparity of race reporting and representation in clinical trials leading to cancer drug approvals from 2008 to 2018. JAMA Oncol 2019;5:e191870. 10.1001/jamaoncol.2019.1870. 31415071 PMC6696743

[4] Ministry of Health, Labour and Welfare , Chiyoda-ku, Tokyo, Japan. Public Assistance Act. https://www.mhlw.go.jp/web/t_doc?dataId=82048000&dataType=0&pageNo=1 (accessed on 1 September 2024).

[5] Ministry of Health, Labour and Welfare , Chiyoda-ku, Tokyo, Japan. Welfare and long-term care public assistance system. 2024. https://www.mhlw.go.jp/stf/seisakunitsuite/bunya/hukushi_kaigo/seikatsuhogo/seikatuhogo/ (accessed on 1 September 2024).

[6] Williams E, Rudowitz R, Garfield R, et al. Medicaid and state financing: Key indicators to watch through pandemic and recovery. San Francisco, CA, United States: Kaiser Family Foundation 2021. https://www.kff.org/medicaid/issue-brief/medicaid-and-state-financing-key-indicators-to-watch-through-pandemic-and-recovery/.

[7] Kahn J, Miller S, Sawant A. As more Americans ask for public aid, could integrated benefits help?. New York, NY, United States: McKinsey & Company 2024. https://www.mckinsey.com/industries/healthcare/our-insights/as-more-americans-ask-for-public-aid-could-integrated-benefits-help.

[8] Wang JY, Wang CY, Juang SY, et al. Low socioeconomic status increases short-term mortality of acute myocardial infarction despite universal health coverage. Int J Cardiol 2014;172:82–7. 10.1016/j.ijcard.2013.12.082. 24444479

[9] Nishioka D, Saito J, Ueno K, Kondo N. Non-financial social determinants of diabetes among public assistance recipients in Japan: A cohort study. J Diabetes Investig 2021;12:1104–11. 10.1111/jdi.13435. PMC8169356 33047513

[10] Carethers M, Doubeni A. Causes of socioeconomic disparities in colorectal cancer and intervention framework and strategies. Gastroenterology 2022;158:354–67. 10.1053/j.gastro.2019.10.029. PMC6957741 31682851

[11] Takahashi T. Management of the corporation based on an assessment of its capacity to provide medical and nursing care. Agency: Welfare And Medical service (WAM), Minato-ku, Tokyo, Japan. October 2015 issue to January 2016 issue. https://www.int.wam.go.jp/sec/com/content/wamnet/pcpub/top/fukushiiryokeiei/houjin/houjin001.html.

[12] Ministry of Health, Labour and Welfare, Chiyoda-ku, Tokyo, Japan. Secondary medical region. 2024. https://www.mhlw.go.jp/file/05-Shingikai-10801000-Iseikyoku-Soumuka/0000127303.pdf.

[13] Uryu K, Imamura Y, Shimoyama R, et al. Stepwise prolongation of overall survival from first to third generation EGFR-TKIs for *EGFR* mutation-positive non-small-cell lung cancer: The Tokushukai REAl-world data project (TREAD 01). Jpn J Clin Oncol 2024;54:319–28. 10.1093/jjco/hyad162. 37997468

[14] Shimoyama R, Imamura Y, Uryu K, et al. Real-world outcome of systemic therapy in Japanese patients with cancer (Tokushkai REAl-world data project: TREAD): Study protocol for nationwide cohort study. Healthcare (Basel) 2022;10:2146. 10.3390/healthcare10112146. 36360487 PMC9690553

[15] National Cancer Registry (Ministry of Health, Labour and Welfare), tabulated by Cancer Information Service, National Cancer Center, Chuo-ku, Tokyo 104-0045, Japan. [Cited Feb 28, 2023]. https://ganjoho.jp/reg_stat/statistics/data/dl/en.html.

[16] Akaike H . Information theory and an extension of the maximum likelihood principle. In: Parzen E, Tanabe K, Kitagawa G, editor. Selected papers of Hirotugu Akaike. Springer, New York, NY: Springer Series in Statistics, 1973; 199–213.

[17] Kraskowski O, Stratmann A, Wiesweg M, et al. Favorable survival outcomes in epidermal growth factor receptor (EGFR)-mutant non-small cell lung cancer sequentially treated with a tyrosine kinase inhibitor and osimertinib in a real-world setting. J Cancer Res Clin Oncol 2023;149:9243–52. 10.1007/s00432-023-04839-3. 37198447 PMC10374675

[18] Manninen O, Puuniemi L, Iivanainen S, et al. Treatment outcomes of non-small cell lung cancers treated with EGFR tyrosine kinase inhibitors: A real-world cohort study. Acta Oncol 2023;62:1854–61. 10.1080/0284186X.2023.2274481. 37934101

[19] Qureshi S, Boily G, Boulanger J, et al. Advanced lung cancer patients’ use of EGFR tyrosine kinase inhibitors and overall survival: Real-world evidence from Quebec, Canada. Curr Oncol 2022;29:8043–73. 10.3390/curroncol29110636. 36354696 PMC9689227

[20] Shenolikar R, Liu S, Shah A, et al. Real-world treatment patterns of metastatic non-small cell lung cancer patients receiving epidermal growth factor receptor tyrosine kinase inhibitors. Cancer Med 2023;12:159–69. 10.1002/cam4.4918. 35702932 PMC9844647

[21] Winfree B, Sheffield M, Cui L, et al. Study of patient characteristics, treatment patterns, EGFR testing patterns and outcomes in real-world patients with *EGFR*m(+) non-small cell lung cancer. Curr Med Res Opin 2022;38:91–9. 10.1080/03007995.2021.1983530. 34544302

[22] Watanabe S, Usui M. Clinical features of ST-segment elevation myocardial infarction in patients receiving welfare public assistance in urban area of Japan. J Cardiol 2021;77:404–7. 10.1016/j.jjcc.2020.10.013. 33183887

[23] Danchin N, Neumann A, Tuppin P, et al. Impact of free universal medical coverage on medical and outcomes in low-income patients hospitalized for acute myocardial infarction: An analysis from the French National Health Insurance system. Circ Cardiovasc Qual Outcomes 2011;4:619–25. 10.1161/CIRCOUTCOMES.111.961193. 21972406

[24] Watanabe T, Rikitake R, Kakuwa T, et al. Time to treatment initiation for six cancer types: An analysis of data from a nationwide registry in Japan. World J Surg 2023;47:877–86. 10.1007/s00268-022-06883-5. 36607390 PMC9821366

[25] Eba J, Nakamura K. Overview of the ethical guidelines for medical and biological research involving human subjects in Japan. Jpn J Clin Oncol 2022;52:539–44. 10.1093/jjco/hyac034. 35349681 PMC9157286

[26] Watanabe K, Sasaki K, Machida R, et al. High-cost treatments for advanced lung cancer in Japan (Lung Cancer Study Group of the Japan Clinical Oncology Group). Jpn J Clin Oncol 2024;54:1084–92. 10.1093/jjco/hyae094. 39158350

