# Correction to: Public assistance and survival equality in patients with *EGFR* mutation-positive lung cancer

**DOI:** 10.1093/jjco/hyag104

**Published:** 2026-06-26

This is a correction to: Kiyoaki Uryu, Yoshinori Imamura, Rai Shimoyama, Takahiro Mase, Yoshiaki Fujimura, Maki Hayashi, Megu Ohtaki, Keiko Otani, Makoto Hibino, Shigeto Horiuchi, Tomoya Fukui, Ryuta Fukai, Yusuke Chihara, Akihiko Iwase, Noriko Yamada, Yukihiro Tamura, Hiromasa Harada, Asuka Tsuya, Takafumi Okabe, Masahiro Fukuoka, Hironobu Minami, Public assistance and survival equality in patients with *EGFR* mutation-positive lung cancer, *Japanese Journal of Clinical Oncology*, Volume 55, Issue 3, March 2025, Pages 228–236, https://doi.org/10.1093/jjco/hyae167

In the originally published version of this manuscript, the funding and grant information was erroneous.

Under the ‘**Funding**’ header, the first sentence should have read: “This work was supported by JSPS KAKENHI Grant Number JP24K13480” instead of: “This work was supported by Health Labour Sciences Research Grant Grant Number 23EA1036”.

These details have been corrected only in this correction notice to preserve the published version of record.

