# Supplementary figures and images for: Public assistance and survival equality in patients with EGFR mutation-positive lung cancer
Source: Jpn J Clin Oncol. 2024 Dec 1;55(3):228–36. doi: 10.1093/jjco/hyae167 (PMC11882504; doi:10.1093/jjco/hyae167)

## Slide 1
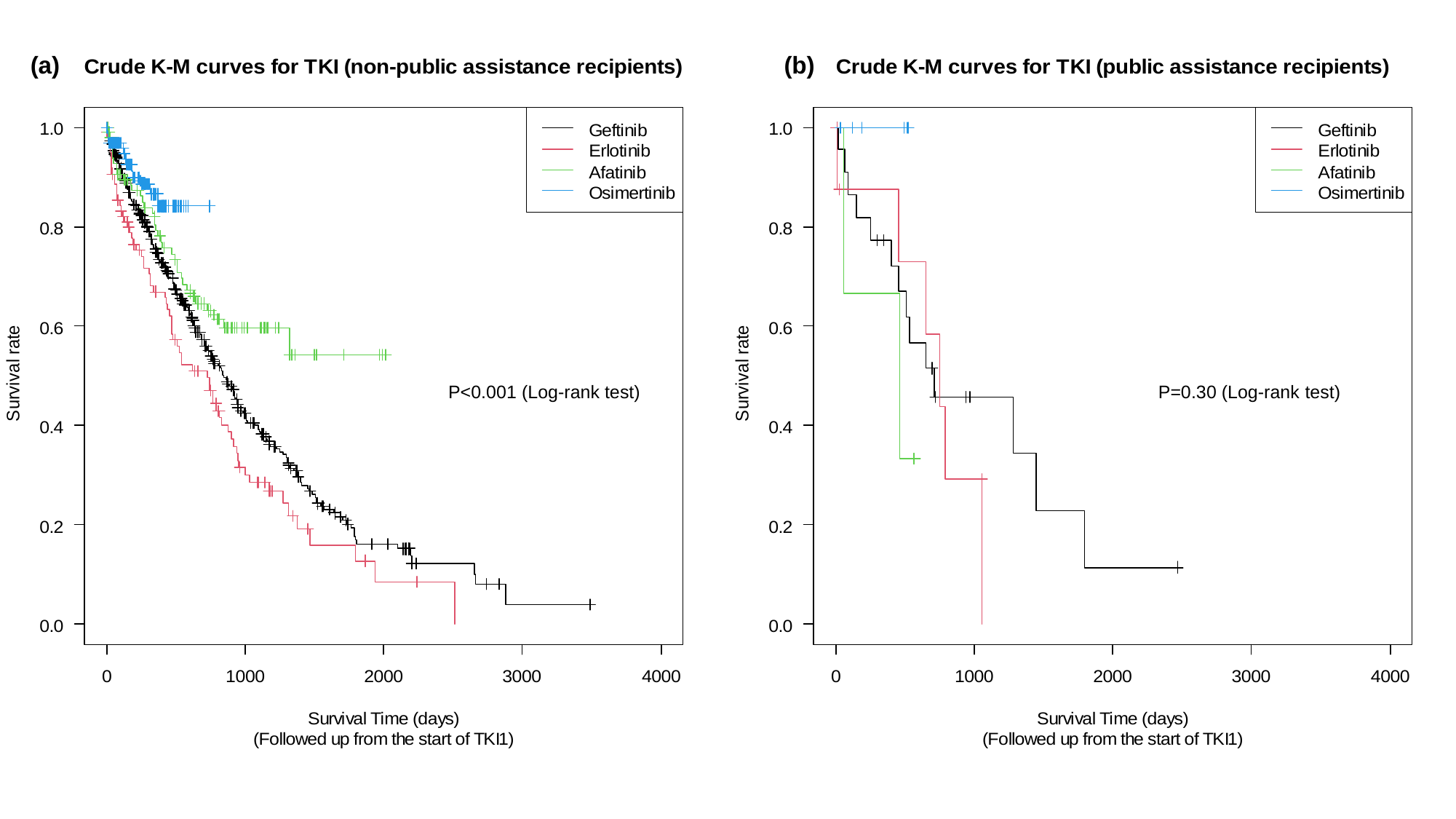

(a)
P<0.001 (Log-rank test)
(b)
P=0.30 (Log-rank test)

Supplement: Figure_S1_2024_11_26_hyae167 [file figure_s1_2024_11_26_hyae167.pptx]
