# Supplementary material for: Public assistance and survival equality in patients with EGFR mutation-positive lung cancer
Source: Jpn J Clin Oncol. 2024 Dec 1;55(3):228–36. doi: 10.1093/jjco/hyae167 (PMC11882504; doi:10.1093/jjco/hyae167)
Supplement: Figure_S2_2024_11_26_hyae167 [file figure_s2_2024_11_26_hyae167.pptx]

## Slide 1
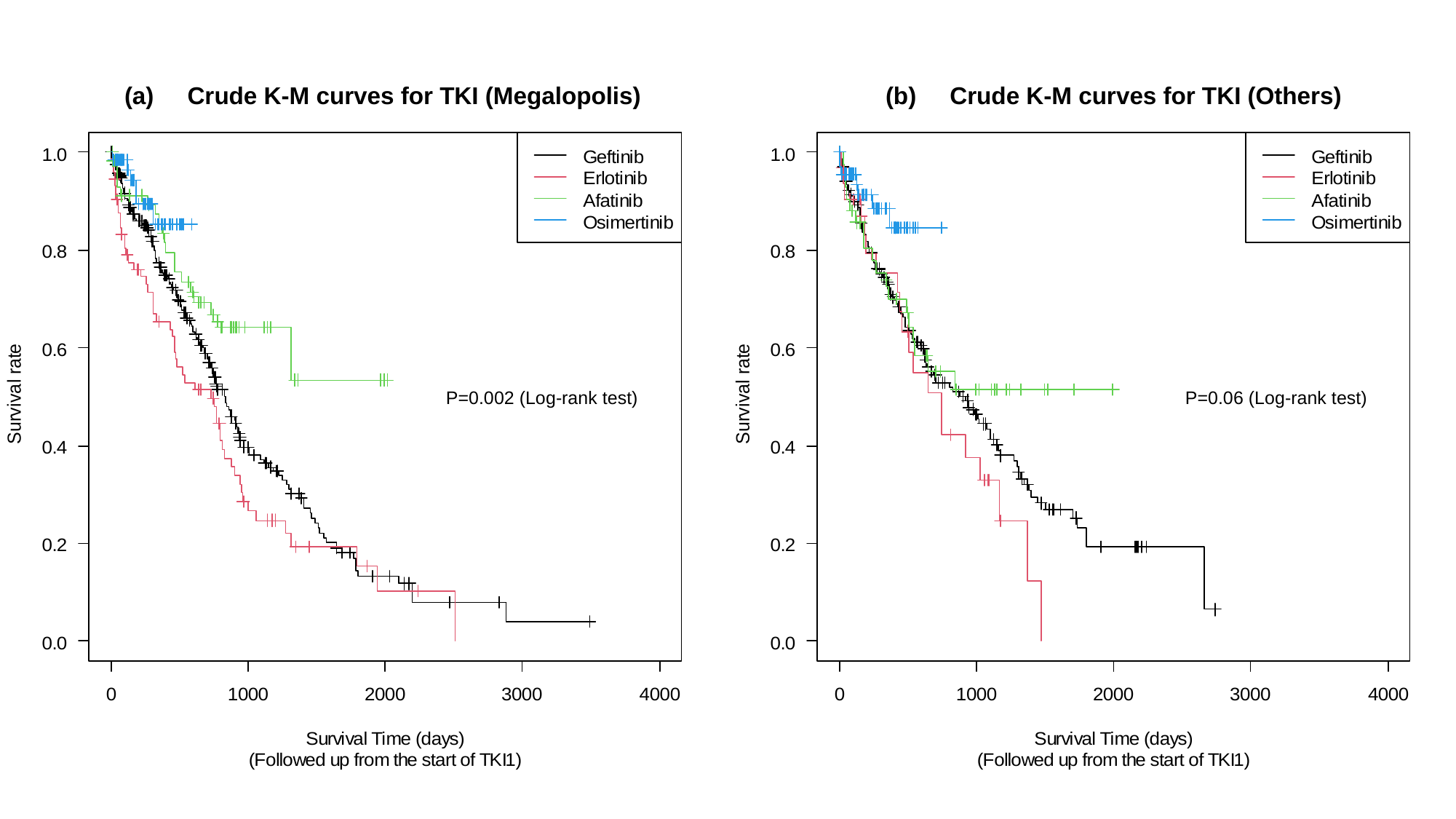

(a) Crude K-M curves for TKI (Megalopolis)
(b) Crude K-M curves for TKI (Others)
P=0.002 (Log-rank test)
P=0.06 (Log-rank test)
