# Supplementary material for: Public assistance and survival equality in patients with EGFR mutation-positive lung cancer
Source: Jpn J Clin Oncol. 2024 Dec 1;55(3):228–36. doi: 10.1093/jjco/hyae167 (PMC11882504; doi:10.1093/jjco/hyae167)
Supplement: Table_S1_2024_11_26_hyae167 [file table_s1_2024_11_26_hyae167.pptx]

## Slide 1
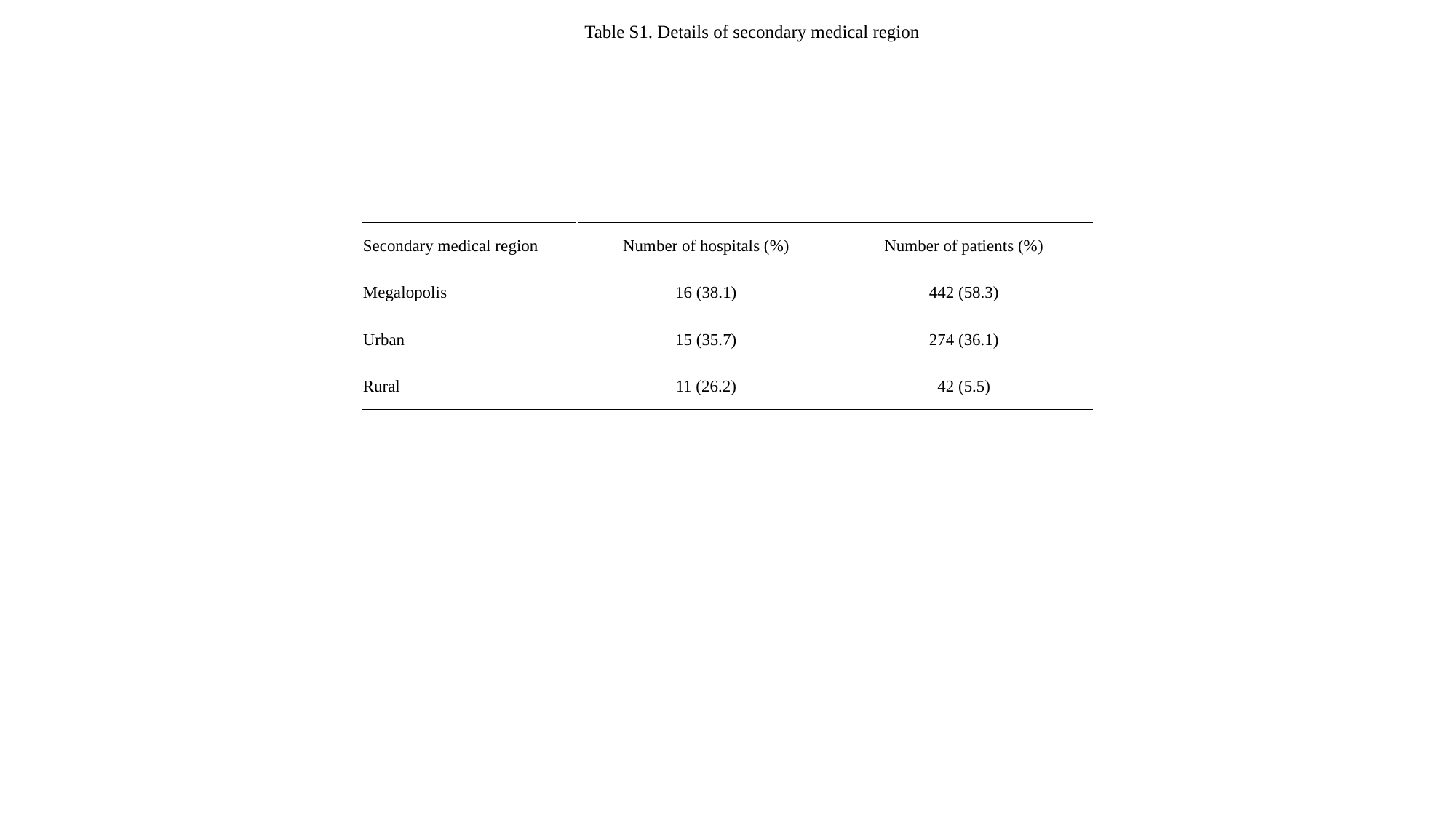

Table S1. Details of secondary medical region
| Secondary medical region | Number of hospitals (%) | Number of patients (%) |
| --- | --- | --- |
| Megalopolis | 16 (38.1) | 442 (58.3) |
| Urban | 15 (35.7) | 274 (36.1) |
| Rural | 11 (26.2) | 42 (5.5) |
